# In vitro anticandidal activity and gas chromatography-mass spectrometry (GC-MS) screening of *Vitex agnus-castus* leaf extracts

**DOI:** 10.7717/peerj.10561

**Published:** 2021-01-04

**Authors:** Ibtisam Mohammed Ababutain, Azzah Ibrahim Alghamdi

**Affiliations:** 1 Basic & Applied Scientific Research Center (BASRC), Imam Abdulrahman Bin Faisal University, Dammam, Saudi Arabia; 2 Department of Biology, College of Science, Imam Abdulrahman Bin Faisal University, Dammam, Saudi Arabia

**Keywords:** Anticandidal activity, Vitex agnus-castus, *Candida albicans*, *Candida tropicalis*, *Candida ciferrii*, GC-MS

## Abstract

**Background:**

Candida infections are becoming more drug resistant; it is necessary to search for alternative medications to treat them. Therefore, the present study estimates the anticandidal activity of *Vitex agnus-castus* (VA-C) leaf extracts.

**Methods:**

We used the agar well diffusion method to assess the anticandidal activity of three different VA-C leaf extracts (ethanol, methanol, and water) against three *Candida* species (*Candida tropicalis*, *Candida albicans*, and *Candida ciferrii*). The minimum inhibitory concentration (MIC) was estimated using the two-fold dilution method and the minimum fungicidal concentration (MFC) was determined using the classic pour plate technique. The MFC/MIC ratio was calculated to estimate the microbicidal or microbiostatic activity. A gas chromatography mass spectrometer was used to screen the phytochemicals of the VA-C leaf extracts (ethanol, methanol, and water).

**Results:**

All VA-C extracts ethanol, methanol, and water were significantly inhibited the growth of the test *Candida* species and the inhibition activity depended on the solvent used and the *Candida* species. The results showed that *C. tropicalis* was the most highly inhibited by all extracts followed by *C. albicans* and *C. ciferrii*. The MIC values were 12.5–25 µg/ml, and MFC values were 25–100 µg/ml. The ratios of MFC/MIC were two-fold to four-fold which was considered candidacidal activity. Ninety-five phytochemical compounds were identified by the GC-MS assay for the VA-C leaf extracts. The total number of compounds per extract differed. Methanol had 43 compounds, ethanol had 47 compounds, and water had 52 compounds. The highest compound concentrations were: 4,5-Dichloro-1,3-dioxolan-2-one in ethanol and methanol, 1H-Indene, 2,3-dihydro-1,1,2,3,3-pentamethyl in ethanol, Isobutyl 4-hydroxybenzoate in methanol, and Benzoic acid and 4-hydroxy- in water. These phytochemical compounds belong to different bioactive chemical group such as polyphenols, fatty acids, terpenes, terpenoids, steroids, aldehydes, alcohols, and esters, and most of which have anticandidal activity.

**Conclusions:**

VA-C leaf extracts may be useful alternatives to anticandidal drugs, based on their effectiveness against all test *Candida* species at low concentrations. However, appropriate toxicology screening should be conducted before use.

## Introduction

The number of severe *Candida* infections is on the rise, which is concerning due to their virulence, ability to survive in extreme environments, and resistance to antifungal agents (Paramythiotou et al., 2014). *Candida* species can cause a wide variety of infections ranging from mild to severe, such as candidemia which has a mortality rate up to 38% in immunosuppressed patients (i.e., organ transplantation patients, patients under chemotherapy, HIV-infected, and diabetic) (Koehler et al., 2019; De Oliveira Santos et al., 2018). The rate of fungal infections, including candidiasis, can reach 20% in the intensive care unit and antifungal medications including azoles, echinocandins, fluoropyrimidines, and polyenes are typically used to treat these infections. However, determining the appropriate dose for treatment is challenging when considering their side effects (Chatelon et al., 2019). Candidiasis is one of the most common fungal diseases in the world and includes cutaneous candidiasis, mucosal candidiasis, onychomycosis, and systemic candidiasis. Healthy individuals are also susceptible to candidiasis (De Oliveira Santos et al., 2018). Genus *Candida* is deuteromycetes fungi and belongs to the Cryptococcaceae family, with up to 200 species. There are thirty species most commonly isolated in human infections including *Candida albicans*, *Candida tropicalis*, *Candida dubliniensis*, *Candida parapsilosis*, *Candida glabrata*, *Candida lusitaniae*, *Candida kefyr*, and *Candida krusei* (Rodrigues, Rodrigues & Henriques, 2019; Kim, Jeon & Jae Kyung Kim, 2016; Brandt & Lockhart, 2012; Miceli, Diaz & Lee, 2011).

Antifungals have a broad range of applications but it is difficult to determine the ideal treatment regime because their use can be limited and is often accompanied by side effects. The indiscriminate use of antibiotics has led to an increased resistance to these types of medications (De Oliveira Santos et al., 2018). Accordingly, researchers are exploring therapeutic alternatives, such as the use of plant essential oils or extracts. These have been proven beneficial in the treatment of several diseases due to their phytochemical components that have physiological and therapeutic effects on humans, limited toxicity, and low therapeutic costs (Abdulrasheed et al., 2019; Sardi et al., 2013). The World Health Organization reports indicate that up to 25% of modern medicines used in the United States of America originate from plants. In Africa and Asia, 80% of the population still uses medicinal herbs in their primary health care centers (World Health Organization, 2002). Moreover, there is documented evidence for the antimicrobial potential of more than 1,340 plants (Yilar, Bayan & Onaran, 2016). *Vitex* is one of the largest of the 250 genera in the family Verbenaceae found worldwide (Ganapaty & Vidyadhar, 2005). The therapeutic applications of *Vitex agnus-castus* (VA-C) and its safety as a medicinal plant are well stated (Niroumand, Heydarpour & Farzaei, 2018; Neves & Da Camara, 2016; Rani & Sharma, 2013). Previous studies have emphasized the antibacterial activity of the essential oils extracted from the seeds and fruit of VA-C (Eryigit et al., 2015; Dervishi-Shengjergji et al., 2014; Ghannadi et al., 2012). Other studies have investigated the antimicrobial activity of essential oils extracted from the leaves of VA-C (Katiraee et al., 2015; Ulukanli et al., 2015). A few studies have demonstrated the antifungal activity of the seed oil (Asdadi et al., 2014). The antifungal potential of VA-C leaves essential oils against plant pathogens (Yilar, Bayan & Onaran, 2016). The antibacterial activity of the leaf extract of VA-C has been identified in a few studies (Ababutain & Alghamdi, 2018; Kalhoro, Farheen & Aqsa, 2014; Arokiyaraj et al., 2009) as well as the antimicrobial activity of VA-C leaf extract (Kalhoro, Farheen & Aqsa, 2014; Maltaş et al., 2010). These studies used only a few bacteria and one *Candida* species (*Candida albican*s). Keikha et al. (2018) evaluated the antifungal activity of ethanolic and aqueous lead extracts on *C. albicans* strains and they found that the ethanol extract was more effective than the aqueous extract against *C. albicans* strains. However, the effect of VA-C leaf extracts of against human *Candida* species has not been well-studied.

Therefore, this study aims to investigate the anticandidal activity and efficiency of VA-C leaf extracts (water, methanol and ethanol) against the three most frequently isolated *Candida* species (*Candida albicans*, *Candida tropicalis* and *Candida ciferrii*). We determined the phytochemicals of these extracts using Gas Chromatography-Mass Spectrometry (GC-MS).

## Materials and Methods

### Plant material

*Vitex agnus-castus* VA-C leaves were collected from a private garden in Dammam City, Saudi Arabia belonging to Ibtisam Mohammed Ababutain. The plant was identified according to Brickell & Zuk (1997).

### Preparation of plant extracts

Vitex agnus-castus leaves were washed with tap water and left to dry for 2 days at room temperature in a well-ventilated room using a fan to speed up the drying process, then ground to a fine powder. Maceration method described by Pandey & Tripathi (2014) was used with little modification, in which 60 g of the leaf powder was transferred to three Erlenmeyer flasks containing 300 ml of the three different solvents: distilled water, methanol (80%), and ethanol (80%) to a final concentration of 20% g/ml. The leaf mixtures were shaken for 72 h at 300 rpm/min/20 °C to extract the active compounds. We used the method previously described in Ababutain (2019) to extract the active compounds as follows: the leaf mixtures were filtered twice, first using Whatman No. 1 filter paper and then using bacterial filters. The filtrates were concentrated in an oven at 80 °C. The residues were re-suspended in dimethyl sulfoxide (DMSO) to a final concentration of 20%. All flasks were kept at 4 °C for further use.

### Agar well-diffusion method

Three different prepared VA-C leaf extracts with a 20% (mg/ml) concentration were screened for their anticandidal activity using the agar well-diffusion method (National Committee for Clinical Laboratory Standards, 1993) against three unicellular fungi. *Candida tropicalis* and *Candida albicans* were provided by King Fahd Hospital, Al Khobar, Kingdom of Saudi Arabia. *Candida ciferrii* was obtained from the Biology Department, College of Science, Imam Abdulrahman Bin Faisal University.

Inoculums of the *Candida* species were prepared from new cultures in potato dextrose broth (PDB). A Biomerieux DensiCHEK plus meter device was used to adjust the cell suspension turbidity at 1–2 × 10^6^ CFU/ml, which represents 0.5 McFarland standards. Each Petri dish was inoculated individually with 0.5 ml of the previous suspension. Melted potato dextrose agar (PDA) was poured over the inoculums, and the plates were rotated to ensure even distribution of the inoculums then left to harden at room temperate for 5 min. Five wells were made on the inoculated PDA using a 6 mm sterile cork-borer. Each well was filled with 100 µL of the plant extracts. Positive and negative controls were included; nystatin (10 mcg) was used as the positive control and DMSO was used as the negative control. The plates were incubated at 37 °C for 24 h. The anticandidal activity of the plant extracts was estimated in millimeters (mm) using a ruler and measuring the free growth zones around the wells. The experiments were performed in three replicates to ensure the reliability of the results.

### Determination of minimum inhibitory concentration

The minimum inhibitory concentration (MIC) of VA-C leaf extracts was estimated using the two-fold dilution method (Omura et al., 1993) as well as the method previously described in Ababutain (2019). Briefly, the plant extracts were diluted with PDB media using 96-well microtiter plates in wells 1–10. Standard *Candida* inoculums at a concentration of 1–2 × 10^6^ CFU/ml were transferred to the wells to make a final concentration of 50%. We used growth media with the *Candida* inoculum in well 11 and growth media with plant extracts in well 12, as positive and negative controls, respectively. The turbidity was examined by the naked eye after an overnight incubation period at 37 °C and the lowest concentration of plant extract showing no *Candida* species growth was recorded as MICs. All experiments were performed in three replicates.

### Determination of minimum fungicidal concentration

The classic pour plate technique was used to determine the minimum fungicidal concentration (MFC) (National Committee for Clinical Laboratory Standards, 1997). Concentrations that showed no *Candida* species growth from previous MIC experiments were transferred to Petri dishes, then 15 ml of melted PDA was poured over it and gently rotated and left to solidify. Inoculated Petri dishes were incubated at 37° for 48 h. The lowest concentration that showed no visible *Candida* species colonies were recorded as MFC (Ababutain, 2019). All experiments were performed in three replicates.

### Determination of anticandidal efficiency

The anticandidal efficiency of VA-C leaf extracts (ethanol, methanol and water) was determined by calculating the ratio of MFC/MIC according to Levison & Levison (2009).

### Gas chromatography-mass spectrometry

We analyzed the bioactive compounds of all three VA-C leaf extracts (ethanol, methanol and water) with a gas chromatography-mass spectrometer (Shimadzu, Kyoto, Japan) model QP2010 SE, with a 5 Sil MS 5% diphenyl/95% dimethyl polysiloxane capillary column (0.25-μm df, 30 meter, 0.25 mmID) using the method previously described in Ababutain (2019). One microliter from each diluted plant extract (100/1,400, V/V in DMSO) was injected individually in the split mode with a split ratio of 1:10. We used the electron impact ionization system at 70 eV ionization energy to determine GC-MS exposure or detection. Pure helium (99.999%) was used as a carrier gas, at a constant column flow 0.7 ml/min and total flow of 10.4 ml/min. The flow control mode had a linear velocity of 29.6 cm/s. The injector temperature was set at 250 °C and the ion-source temperature was set at 250 °C. The column temperature was programed at 50–300 °C, with a hold time of 3 min, and a total run time of 29 min. The chemical compounds were identified using the National Institute of Standards and Technology (NIST 08) library match and the quantitative data were generated automatically as a percentage (Adams, 2007).

### Statistical analysis

The anticandidal activity of the VA-C leaf extract between the solvents and the *Candida* species was conducted using one-way ANOVA test. A *P*-value of <0.01 was considered statistically significant. Statistical data were analyzed using Statistical Packages for Software Sciences (Statistical Packages for Software Sciences, 2013) version 21 Armonk, New York, IBM Corporation.

## Results

### Anticandidal activity of VA-C leaf extracts

The VA-C extracts were shown to inhibit the growth of all tested *Candida* species and the inhibition activity depended on the solvent type and *Candida* species. The results showed that *C. tropicalis* was the most inhibited by all the extracts followed by *C. albicans* and *C. ciferrii* (all *P* = 0.01). The effects of the ethanol extract against *C. tropicalis*, *C. albicans* and *C. ciferrii* were significantly higher compared to water and methanol extracts at *P* = 0.01, *P* = 0.037 and *P* = 0.047, respectively (Table 1).

**Table 1:** Anticandidal activity of VAC leaves extract at concentration of 20% by using well diffusion assay.

*Candida* species	Zone of inhibition (mm) Mean ± SD					
	Nystatin (10 mcg)	Negative control	Ethanol	Water	Methanol	*P*-value*
*C. tropicalis*	11.0 ± 1.00	0	7.50 ± 0.50	5.67 ± 0.29	5.33 ± 0.29	0.01**
*C. albicans*	5.83 ± 0.29	0	5.83 ± 0.29	5.00 ± 0.50	5.00 ± 0.50	0.047**
*C. ciferrii*	ND	0	4.33 ± 0.58	3.33 ± 0.29	3.33 ± 0.29	0.037**
*P*-value	0.01**	–	0.01**	0.01**	0.01**	–

**Notes:**

* *P*-value has been calculated using one-way ANOVA.

** Significant at *p* < 0.01 level. ND, not identified.

Minimum inhibitory concentration results were between 12.5 µg/ml and 25 µg/mL and all extracts showed similar activity against all *Candida* species at MIC 25 µg/ml, except *C. tropicalis* which was the most sensitive to the ethanol extract at MIC 12.5 µg/ml. The MFC results were between 25 µg/ml and 100 µg/ml. Most extracts showed similar MFC values against all *Candida* species at MFC 50 µg/mL except *C. tropicalis*. The MFC ethanol extract at 25 µg/ml had the highest anticandidal activity against *C. tropicalis* and the MFC methanol extract at 100 µg/ml was considered to be the lowest anticandidal activity against *C. albicans*. The results revealed that both MIC and MFC values for all three solvents were narrow where the differences between values were one to two concentrations only. The MFC/MIC ratio in all the three extracts were only two-fold to four-fold, which means that VA-C leaf extracts are potentially candidacidal (Table 2).

**Table 2:** Minimal Inhibitory Concentration (MIC) µg/ml and Minimal Fungicidal Concentration (MFC) µg/ml and their ratio of VA-C leaves extracts.

*Candida* species	Ethanol			Water			Methanol		
	MIC	MFC	Ratio*	MIC	MFC	Ratio*	MIC	MFC	Ratio*
*C. tropicalis*	12.5	25	2	25	50	2	25	50	2
*C. albicans*	25	50	2	25	50	2	25	100	4
*C. ciferrii*	25	50	2	25	50	2	25	50	2

**Note:**

* Ratio MFC/MIC.

### Gas chromatography-mass spectrometry analysis

Our results revealed that VA-C leaf extracts are rich in phytochemical components of different concentrations. A total of 95 chemical compounds were extracted depending on the solvent type and, of these, 13 compounds were extracted by all three solvents and the total number of extracted compounds was 52 by water extraction, 47 by ethanol extraction, and 43 by methanol extraction (Table 3).

**Table 3:** GC-MS analysis of VA-C leaves extracts, their molecular formula, nature and biological activities.

No	Compound name	Peak area %			Molecular formula	Compound nature and biological activities
		Ethanol	Methanol	Water		
1	4,5-Dichloro-1,3-dioxolan-2-one	7.43	7.45	1.39	C_3_H_2_Cl_2_O_3_	No report was found
2	Benzoic acid, 4-hydroxy-	2.13	3.95	5.99	C_7_H_6_O_3_	Phenolic compounds (Eseyin et al., 2018)
3	5-Hydroxymethylfurfural	1.18	1.61	0.82	C_6_H_6_O_3_	Organic compound Antioxidant and Antiproliferative (Ibrahim, Ali & Zage, 2016)
4	Phenol	1.12	1.73	1.41	C_6_H_5_OH	Phenolic compound, antiviral, antibacterial and antifungal activities (Özçelik, Kartal & Orhan, 2011)
5	4H-Pyran-4-one, 2,3-dihydro-3,5-dihydroxy-	0.75	0.82	1.41	C_6_H_8_O_4_	Flavonoids, Anti-inflammatory, analgesic, antimicrobial activity (Neeraj, Vasudeva & Sharma, 2019)
6	Catechol	0.50	0.57	1.14	C_6_H_4_(OH)_**2**_	Polyhydric phenol, antiviral, antimicrobial activities (Özçelik, Kartal & Orhan, 2011)
7	Benzeneacetaldehyd, alpha-methyl-	0.35	0.65	1.26	C_9_H_10_O	Hydrotropic aldehyde
8	Benzeneacetic acid, 4-hydroxy3-methoxy,	0.39	0.38	0.58	C_10_H_12_O_4_	No report was found
9	Pentanal	0.10	0.16	0.88	C_5_H_10_O	alkyl aldehyde, Inhibition bacteria (Lamba, 2007)
10	Squalene	0.13	0.37	0.40	C_30_H_50_	Terpenoid, Anticandidal activity, antioxidant, anti-inflammatory, and anticancer agent (Ghimire et al., 2016; Zore et al., 2011)
11	Maltol	0.06	0.14	0.77	C_6_H_6_O_3_	Antimicrobial activity (Saud, Pokhrel & Yadav, 2019)
12	1H-Benzocyclohepten-7-ol,2,3,4,4a,5,6,7,8-	0.36	0.12	0.11	C_15_H_26_O	Sesquiterpenids (Solanki, Singh & Sood, 2018)
13	n-Hexadecanoic acid	0.70	0.56	0.96	C_16_H_32_O_2_	Palmitic saturated Fatty acid ester, antimicrobial, antitumor activities, antioxidant, pesticide, nematicide, antiandrogenic and hypochloesterolemi (Tyagi & Agarwal, 2017; Karthikeyan et al., 2014; Sermakkani & Thangapandian, 2012;)
14	3,5-Octadienoic acid, 7-hydroxy-2-methyl	0.85	1.04	–	C_9_H_14_O_3_	No report was found
15	Eugenol	0.39	0.36	–	C_10_H_12_O_2_	Phenolic compounds, antimicrobial activity, insecticide nematicide and food additive (Tan & Nishida, 2012; Johny et al., 2010)
16	1,2,3-Benzenetriol	0.56	0.67	–	C_6_H_6_O_3_	No report was found.
17	Propylphosphonic acid, di(2-ethylhexyl) ester	2.57	1.18	–	C_21_H_40_O_4_	Ester
18	Methylparaben	0.39	0.28	–	C_8_H_8_O_3_	Antimicrobial activity, food preservative, added to cosmetic products, and pharmaceutical products (Mincea et al., 2009)
19	1H-Cycloprop[e]azulen-7-ol, decahydro-1,1,7-trimethyl-4-methylene	1.21	1.04	–	C_15_H_24_O	No report was found
20	Triacetin	0.19	0.27	–	C_9_H_14_O_6_	Triester of glycerin and acetic acid
21	5-(1-Isopropenyl-4,5-dimethylbicyclo [4.3.0]	1.13	0.87	–	C_22_H_36_O_2_	No report was found
22	2,4-Cholestadien-1-one	1.72	0.96	–	C_27_H_42_O	No report was found
23	Phytol	3.31	1.81	–	C_20_H_40_O	Diterpene, antiviral and antimicrobial activities (Özçelik, Kartal & Orhan, 2011)
24	9,12,15-Octadecatrienoic acid, (Z, Z,Z)-	1.18	0.90	–	C_18_H_30_O_2_	Linolenic Omega-3 polyunsaturated fatty acid, anti–inflammatory (Sermakkani & Thangapandian, 2012)
25	Cedran-diol, (8S,14)-	0.13	0.52	–	C_15_H_26_O_2_	No report was found
26	3,7,11,15-Tetramethyl-2-hexadecen-1-ol	1.11	1.34	–	C_20_H_40_O	Terpene Alcohol, antimicrobial, antioxidant, anti–inflammatory and flavoring agent (Shibula & Velavan, 2015; Jegadeeswari et al., 2012; Sermakkani & Thangapandian, 2012)
27	Spiro [4.5] dec-9-en-1-ol,1,6,6,10-tetramethyl	0.54	0.39	–	C_14_H_24_O	No report was found
28	Dodeca-1,6-dien-12-ol, 6,10-dimethyl	1.57	1.57	–	C_14_H_26_O	No report was found
29	Octadecanoic acid	0.51	–	0.22	C_17_H_35_CO_2_H	Stearic saturated fatty acid
30	Benzenediazonium, 2-hydroxy-, hydroxide, i	0.86	–	1.17	C_6_H_5_N_2_O	No report was found
31	Vitamin E	–	0.39	0.36	C_29_H_50_O_2_	Lipid, antibacterial, anti-alzheimer, antiaging and antioxidant (Kumaravel, Muthukumaran & Shanmugapriya, 2017; Al-Marzoqi, Hadi & Hameed, 2016; Shahina et al., 2016; Al-Salih et al., 2013)
32	Cedrol	–	0.22	0.12	C_15_H_26_O	sesquiterpene alcohol
33	gamma-Sitosterol	–	0.89	0.56	C_29_H_50_O	Steroid, antidiabetic drug (Tripathi et al., 2013)
34	Paromomycin	–	0.12	0.22	C_23_H_45_N_5_O_14_	Treatment of diarrhea and protozoa infections (Olajuyigbe et al., 2018)
35	Heptanal	0.20	–	–	C_7_H_14_O	aldehyde antibacterial activity (Lamba, 2007)
36	Ionone	0.33	–	–	C_13_H_20_O	Sesquiterpenoids, antimicrobial agents (Sharma et al., 2012)
37	Chloroxylenol	0.16	–	–	C_8_H_9_OCl	phenols with antiseptic activity, It is used in the manufacture of disinfectants and sterilizers (McDonnell, 2009)
38	1-Heptadecene	0.22	–	–	C_17_H_34_	unsaturated aliphatic hydrocarbons
39	Undecanal	0.25	–	–	C_10_H_21_CHO	fatty aldehyde lipid molecule
40	1H-Indene, 2,3-dihydro-1,1,2,3,3-pentamethyl	9.63	–	–	C_14_H_20_	No report was found
41	Epiglobulol	0.97	–	–	C_15_H_26_O	Alcohol
42	tau-Cadinol	1.64	–	–	C_15_H_26_O	No report was found
43	alpha-Cadinol	0.38	–	–	C_15_H_26_O	Antifungal activity (Cheng et al., 2012)
44	Phytol, acetate	0.37	–	–	C_22_H_42_O_2_	Food additive, antimicrobial, anti-inflammatory, anticancer and antidiuretic properties (Sermakkani & Thangapandian, 2012)
45	1S,2S,5R-1,4,4-Trimethyltricyclo [6.3.1.0(2,5)	1.26	–	–	C_15_H_24_	No report was found
46	beta-iso-Methyl ionone	0.39	–	–	C_14_H_22_O	No report was found
47	Longipinane, (E)-	0.41	–	–	C_15_H_24_	No report was found
48	(-)-Isolongifolol, methyl ether	0.70	–	–	C_16_H_28_O	Ether
49	Taraxasterol	0.07	–	–	C_30_H_50_O	Anti-tumor and chemopreventive activity (Ovesná, Vachálková & Horváthová, 2004)
50	S-Methyl methanethiosulphonate	0.07	–	–	CH_3_SO_2_SCH_3_	Ester, Antimutagenic agent and antimicrobial activity (Joller et al., 2020; Miguel et al., 2016)
51	1-Heptatriacotanol	0.23	–	–	C_37_H_76_O	Fatty alcohol
52	2-Vinylfuran		0.83		C_6_H_6_O	Antimicrobial activity (Drobnica & Sturdík, 1980)
53	Salicyl hydrazide	–	0.39	–	C_7_H_8_N_2_O_2_	Phenolic compounds, antimicrobial activity, Anti-inflammatory (Madan & Levitt, 2014)
54	Isobutyl 4-hydroxybenzoate	–	8.91	–	C_11_H_14_O_3_	No report was found
55	Methyl(ethenyl)bis(but-3-en-1-ynyl) silane	–	1.62	–	C_7_H_16_Si	No report was found
56	beta Carotene	–	0.16	–	C_40_H_56_	Carotenoids used as food, nutrition, antioxidant, disease control, and antimicrobial agents (Kirti et al., 2014)
57	17-Norkaur-15-ene, 13-methyl-, (8.beta.,13.b	–	1.05	–	C_20_H_32_	No report was found
58	3-Hydroxy-2-(2-methylcyclohex-1-enyl) propan-	–	1.48	–	C_10_H_16_O_2_	No report was found
59	Cyclopropanebutanoic acid, 2-[[2-[[2-[(2-pen	–	1.20	–	C_11_H_22_N_2_O_4_	No report was found
60	Cholan-24-oic acid, methyl ester, (5.beta.)-	–	1.56	–	C_25_H_40_O_3_	No report was found
61	Lup-20(29)-en-3-ol, acetate, (3.beta.)-	–	1.12	–	C_32_H_52_O_2_	No report was found
62	geranyl-.alpha.-terpinene	–	0.80	–	C_20_H_32_	Terpinene
63	Tungsten, tricarbonyl-(2,5-norbornadiene)	–	–	1.32	C_14_H_16_	No report was found
64	1,2-Cyclopentanedione	–	–	1.21	C_7_H_10_O_2_	Prevents gastrointestinal tumor growth (Neeraj, Vasudeva & Sharma, 2019)
65	2-Cyclopenten-1-one, 2-hydroxy-3-methyl-	–	–	0.34	C_6_H_8_O_2_	No report was found
66	1,2,3-Propanetriol, 1-acetate	–	–	1.23	C_5_H_10_O_4_	No report was found
67	Acetoacetic acid, 3-thio-, benzyl ester	–	–	0.16	C_11_H_12_O_2_	No report was found
68	trans-Z-.alpha.-Bisabolene epoxide	–	–	1.11	C_15_H_24_O	No report was found
69	2-Hydroxyoctanoic acid	–	–	0.62	C_8_H_16_O_3_	No report was found
70	1-Tetradecene	–	–	0.67	C_14_H_28_	Antimicrobial activity (Naragani et al., 2016)
71	Benzoic acid, 4-methoxy-	–	–	0.69	C_8_H_8_O_3_	No report was found
72	Chlorozotocin	–	–	0.20	C_9_H_16_ClN_3_O_7_	No report was found
73	2-Isopropyl-5-methyl-6-oxabicyclo [3.1.0] hex	–	–	1.51	C_10_H_16_O_2_	No report was found
74	Quinic acid	–	–	2.96	C_7_H_12_O_6_	Anti-viral activity (Özçelik, Kartal & Orhan, 2011)
75	3-Methylindene-2-carboxylic acid	–	–	1.11	C_11_H_10_O_2_	No report was found
76	O, O-Dibutyl S-(2-acetamidoethylmercapto) p	–	–	1.32	C_12_H_22_O_4_	No report was found
77	3-Deoxy-d-mannonic acid	–	–	1.21	C_6_H_12_O_6_	No report was found
78	Cyclooctane-1,4-diol, cis	–	–	0.44	C_8_H_16_O_2_	No report was found
79	cis, cis, cis-7,10,13-Hexadecatrienal	–	–	0.58	C_16_H_26_O	Unsaturated fatty aldehyde
80	10-Iodo-7-oxa-2-thia-tricyclo [4.3.1.0(3,8)]de	–	–	1.08	C_8_H_11_IOS	No report was found
81	Bicyclo [6.1.0] nonane, 9-(1-methylethylidene	–	–	3.66	C_12_H_20_	No report was found
82	Inositol	–	–	0.15	C_6_H_12_O_6_	Essential nutrient, Cancer chemoprevention agent, treatment for Polycystic Ovary Syndrome and insulin sensitizing agent (Carlomagno & Unfer, 2011)
83	Xylose	–	–	0.15	C_5_H_10_O_5_	Pentose sugar (Huntley & Patience, 2018)
84	Scyllo-Inositol	–	–	1.18	C_6_H_12_O_6_	treatment of Alzheimer’s disease (Ma, Thomason & McLaurin, 2012)
85	2,4-Pentadien-1-ol, 3-pentyl-, (2Z)-	–	–	0.94	C_10_H_18_O	No report was found
86	Widdrol hydroxyether	–	–	0.23	C_15_H_26_O_2_	No report was found
87	Stigmasterol	–	–	0.21	C_29_H_48_O	Steroid, antioxidant, antimicrobial, anticancer, antiarthritic, antiasthma, anti-inflammatory, diuretic (Tyagi & Agarwal, 2017; Kumar, Vasantha & Mohan, 2014)
88	beta.-Amyrin	–	–	0.43	C_30_H_50_O	Triterpenes, anti-inflammatory (Okoye et al., 2014)
89	5,5′-Dihydroxy-3,3′-dimethyl-2,2′-binaphthal	–	–	1.31	C_17_H_14_O_6_	No report was found
90	Lanosterol	–	–	0.22	C_30_H_50_O	Sterol, essential components of eukaryotic cells (Wei, Yin & Welander, 2016)
91	Betulin	–	–	0.58	C_30_H_50_O_2_	Anti-Viral and anti-tumour (Tolstikov et al., 2005)
92	alpha-Tocopheryl acetate	–	–	0.23	C_31_H_52_O_3_	Antimicrobial activity (Bidossi et al., 2017)
93	Geldanamycin	–	–	0.33	C_29_H_40_N_2_O_9_	Chemotherapeutic agents (Da Rocha, Lopes & Schwartsmann, 2001)
94	Dihydrosteviobiside	–	–	0.26	C32H52O13	No report was found
95	Bronopol	–	–	0.10	C_3_H_6_BrNO_4_	Antimicrobial activity (Birkbeck et al., 2006; Treasurer, Cochrane & Grant, 2005)
Total compounds for each solvent		47	43	52		

## Discussion

Antibiotic resistance is becoming more common among a larger number of microorganisms, including *Candida* species, leading to a heightened interest in finding alternative treatments. The secondary metabolites of plants have made them useful for treating a variety of diseases, flavoring foods and products, preserving food, in pesticides, in perfumes and cosmetics, and more recently to inhibit the microbial growth. VA-C leaf extracts have been reported to cause mild and reversible side effects such as headache, acne, nausea, gastrointestinal disturbances, erythematous rash, pruritus, and menstrual disorders. However, no drug interactions have been associated with VA-C leaf extracts (Daniele et al., 2005). Therefore, VA-C leaf extracts (ethanol, methanol, and water) were investigated for their ability to inhibit the growth of three Azoles antibiotic-resistant *Candida* species: *C. ciferrii*, *C. albicans*, and *C. tropicalis* (Romald et al., 2019; Bhakshu, Ratnam & Raju, 2016).

Our results showed that alcohol extracts (methanol and ethanol) and aqueous extract have the ability to inhibit the growth of all tested *Candida* species. These results are in agreement with Kalhoro, Farheen & Aqsa (2014) study, which found that the ethanol VA-C leaf extract has the potential to inhibit the growth of *C. albicans*. Our results are also consistent with Maltaş et al. (2010) who observed that the methanol extract of VA-C leaves inhibits the growth of *C. albicans*. Moreover, we showed that the inhibitory capacity of the solvents varied significantly in descending order of ethanol, then water, then methanol. These results are in line with a recent study conducted by Keikha et al. (2018) who found that VA-C ethanol leaf extract has the highest inhibiting effect against *C. albicans* isolates than water extract. Our results showed a similarity in the inhibitory effect of all extracts with nystatin (10 mcg) as a positive control against *C. albicans*, where the inhibitory effect for the positive control was higher than all extracts against *C. tropicalis*.

We found that MIC showed that the ethanol extracts of VA-C were relatively higher than water and methanol. MIC values were between 12.5 µg/ml and 25 µg/ml for ethanol and represented a dilution of 4 and 3, respectively. For water and methanol the MIC values are specified at 25 µg/ml which represents dilution 3. Our results are similar to those of Keikha et al. (2018) who also found that the ethanol extract of VA-C was more effective than the aqueous extract when the MIC values of ethanol against isolates of *Candida* species were between 0.78 µg/ml and 1.56 µg/ml, which represent dilution 7 and 8, respectively. The values of the aqueous extract were between 6.25 µg/ml and 1.562 µg/ml, which represent dilutions of 5 and 7, respectively.

There was a convergence of MFC values, which represents only the three dilutions from 1 to 3 (100 µg/ml and 25 µg/ml), respectively. The VA-C extract of ethanol was the most influential on *C. tropicalis* with the value of MFC 25 µg/ml and the aqueous extract was less effective on *C. albicans*, with a value of 100 µg/ml.

Selection of antibiotics for the treatment of infections is highly influenced by the mechanism of action. Antibiotics classified into either by killing the microbe (microbicidal) or inhibiting its growth (microbistatic) (Etebu & Arikekpar, 2016). Antibiotics with inhibitory effects are usually prescribed to patients who do not have problems with their immune system, while antibiotics with a fatal effect are prescribed for patients with low immunity or severe infections (Davies & Davies, 2010). *Candida* species are generally opportunistic and affect the group of people with low immunity so antibiotics that are prescribed are generally more effective if they are of the fatal type. Therefore, the inhibitory efficiency of the VA-C extract was estimate using the ratio between MFC and MIC. Our results showed that the ratio of MFC/MIC between two-fold to four-fold have a candidacidal effect (Levison & Levison, 2009). To our best of our knowledge, ours is the first study to establish this finding.

We found that the extracts of VA-C differed in their inhibitory effect according to the type of solvent and this is may be due to the difference in the degree of polarity between the solvent. Water has the highest polarity of 1,000 followed by methanol (0.762) and finally, ethanol (0.654). The compounds extracted by these highly polar solvents differ in quantity and quality (Abubakar & Haque, 2020). Many studies have demonstrated the effect of the solvent type on the inhibitory potential of plant extracts (Aljuraifani, 2017; Ababutain, 2015).

The GC-MC analysis result revealed that all three VA-C extracts were rich in chemical compounds that act as an anti-inflammatory, anticancer, anti-Alzheimer, anti-diarrheal, anti-diabetic, anti-viral, antioxidant, anti-allergic, nematicide, antibacterial, antifungal. These extracts are also used as food preservatives and flavorings, as previously found in other published works (Table 3). Several of these secondary metabolites belong to important chemical groups such as polyphenols, fatty acids, terpenes, terpenoid, steroids, aldehydes, alcohol, and esters. These results are in agreement with a previous study of Keikha et al. (2018), which stated that the VA-C extract was rich in chemical compounds, and the alcoholic extract contained 36 chemical compounds that belong to different chemical groups. Our results showed that the majority of compounds were 4,5-Dichloro-1,3-dioxolan-2-one in both ethanol and methanol, 1H-Indene, 2,3-dihydro-1,1,2,3,3-pentamethyl in ethanol, Isobutyl 4-hydroxybenzoate in methanol, and Benzoic acid and 4-hydroxy- in water. Keikha et al. (2018) found that the majority of compounds in the VA-C ethanol extract were α-Pinene, isoterpinolene, caryophyllene, and azulene. The difference in the number of phytochemical compounds may be attributed to the variations among the VA-C genotypes (Karaguzel & Girmen, 2009).

The inhibitory activity of VA-C extracts maybe attributed to the presence of important bioactive compounds (Abdal Sahib, Al-Shareefi & Hameed, 2019), which may target different structures of the *Candida* species including the cell wall, cell membrane, and mitochondria enzymes. Some of these compounds may reduce or prevent the virulence factors, including adhesins, enzymes production, germ tubes (Pseudohyphal), biofilm formation, and quorum sensing (De Oliveira Santos et al., 2018; Liu et al., 2017; Sardi et al., 2013). Our results showed the diversity of the compounds extracted from VA-C plant leaves that belong to several effective biochemical compounds with different anticandidal activity, including polyphenols that can destroy the *Candida* cell membrane leading to permeability of the cell contents (Peralta et al., 2015; Hwang et al., 2011; Hwang et al., 2010), inhibition of mitochondrial enzyme activity in the *Candida* cell (Yang et al., 2014) and inhibition of the germ tube formation (Seleem et al., 2016). Fatty acids with carbon chains between 10 and 12 carbons had a good inhibitory effect against *Candida* species (Ababutain, 2019; Bergsson et al., 2001). Terpenes have been reported to have inhibitory effects against *C. albicans* and may prevent biofilm formation (Pemmaraju et al., 2013). Terpenoids inhibit *C. albicans* cell growth by affecting the membrane and preventing adhesins, biofilm formation, and germ tube formation (Touil et al., 2020; Raut et al., 2013; Zore et al., 2011).

## Conclusions

Our results showed that VA-C leaf extract is rich in bioactive compounds with broad spectrum activity that inhibited all the tested *Candida* species despite different species. Accordingly, VA-C leaf extracts may inhibit the growth of *Candida* species in general, compared to antifungals that affect a specific species or a strain of species and require an accurate diagnosis of the *Candida* isolation to choose the appropriate antifungal. The inhibitory activity of the ethanol solvent was better than methanol and water, which may indicate the importance of choosing the appropriate solvent to extract phytochemicals with high inhibiting effectiveness and in higher quantities. Moreover, our results showed that the extract had a candidacidal effect on test *Candida* species at low concentrations, which may reduce the side effects of the extract. VA-C leaf extracts are advantageous, and a promising component that can be used to develop an alternative anticandidal agent. Further studies are required to assess the toxicity, genotoxicity and mutagenicity of VA-C extracts and prove their safety for human use.

## Supplemental Information

10.7717/peerj.10561/supp-1 Supplemental Information 1 Anticandidal activity of VAC leaves extract at concentration of 20% by using well diffusion assay.

10.7717/peerj.10561/supp-2 Supplemental Information 2 Minimal Inhibitory Concentration (MIC) µg/ml and Minimal Candidacidal Concentration (MFC) µg/ml and their ratio of VA-C leaves extracts.

## Additional Information and Declarations

### Data Availability

The following information was supplied regarding data availability: Raw data are available as Supplemental Files.
